# Confrontation Naming and Reading Abilities at Primary School: A Longitudinal Study

**DOI:** 10.1155/2015/981548

**Published:** 2015-06-01

**Authors:** Chiara Luoni, Umberto Balottin, Laura Rosana, Enrico Savelli, Silvia Salini, Cristiano Termine

**Affiliations:** ^1^Child Neuropsychiatry Unit, Department of Clinical and Experimental Medicine, University of Insubria, Varese, Italy; ^2^Department of Child Neurology and Psychiatry, IRCCS “C. Mondino” Foundation, University of Pavia, Pavia, Italy; ^3^Department of Education, University of San Marino, Montegiardino, San Marino; ^4^Data Science Lab, Department of Economics, Management and Quantitative Methods, University of Milan, 2012 Milan, Italy

## Abstract

*Background*. Confrontation naming tasks are useful in the assessment of children with learning and language disorders. *Objectives*. The aims of this study were (1) providing longitudinal data on confrontation naming; (2) investigating the role of socioeconomic status (SES), intelligence, age, and gender in confrontation naming; (3) identifying relationship between confrontation naming and reading abilities (fluency, accuracy, and comprehension). *Method*. A five-year longitudinal investigation of confrontation naming (i.e., the Boston Naming Test (BNT)) in a nonclinical sample of Italian primary school children was conducted (*n* = 126), testing them at the end of each school year, to assess nonverbal intelligence, confrontation naming, and reading abilities. *Results*. Performance on the BNT emerged as a function of IQ and SES. Significant correlations between confrontation naming and reading abilities, especially comprehension, were found; BNT scores correlated better with reading fluency than with reading accuracy. *Conclusions*. The longitudinal data obtained in this study are discussed with regard to reading abilities, intelligence, age, gender, and socioeconomic status.

## 1. Introduction

Impaired rapid naming, along with poor phonological processing, is considered a core deficit in reading-impaired children and many studies have found correlations between rapid automatic naming (RAN) tasks and reading abilities [1–4]. But confrontation naming tasks, too, in particular the Boston Naming Test (BNT), could also be useful in the assessment of children with learning and language disorders [5, 6]: correlations have been found between BNT scores and reading performances, particularly reading comprehension [7].

In fact, there is a growing body of evidence suggesting that naming skill is deeply rooted in phonological competence and language-related abilities: both rapid automatic and discrete naming have been found to discriminate reliably between good and poor readers at different ages, from preschool [8–10] to adulthood [11]; furthermore, because of its persistence, even long after a reading deficit has been compensated for, impaired naming has been considered one of the main symptoms of the phonological “core” weakness in developmental dyslexia. In this regard, further evidence is provided by studies that have found impaired naming in poor readers even compared to reading age-matched controls [12]. Moreover, Swan & Goswami [13] found that while both developmental dyslexic (DD) and “garden variety” (GV) poor readers performed significantly more poorly on picture naming tasks than both chronological age- and reading age- matched controls, only the DD group performed more poorly on word length- and word-frequency related tasks, a finding suggested to indicate the presence of a specific weakness in the degree of specification of phonological representations and in their retrieval. Indeed, when the authors compared the performances of these two groups, they found that whereas poor performance in the GV group stemmed from a lack of vocabulary knowledge, in the DD group it was caused by a genuine word retrieval deficit (i.e., the participants had the word in their receptive vocabulary but were unable to access it). Similar results have been obtained in German children [14], lending further support to the notion of a basic phonological weakness, particularly in more transparent orthographies. More recently, however, three Italian [15–17] found a stronger relationship of rapid naming ability (as opposed to phonological awareness skills) with reading, a result in line with the findings of a previous study by Wimmer et al. [18]. These data throw into question the simple idea of a basic phonological deficit underlying both naming and reading ability, at least in transparent orthographies, and emphasise the potential role of other psycholinguistic and visual-attentional variables, assumed to be involved in RAN [19].

To date, only a few studies on naming (confrontation and/or rapid) and its relationships with reading have been conducted in children learning transparent orthographies (German, Italian, Spanish, Greek, etc.) and their results, as regards the different components underlying naming (rapid and/or confrontation) [20] and its possible specific relationship with peculiarities of different orthographies [21, 22], have been interpreted controversially. However, it nevertheless seems reasonable to conclude that they point to a common deficit in accessing phonological representations [23], a deficit whose contribution may change in relation to the demands, in literacy acquisition and reading, posed by different orthographic systems.

On the other hand, it should be emphasized that intelligence level and home literacy environment are also factors that influence naming performance [24, 25]. Moreover, it has been shown that age is an important variable not only for naming but also for reading abilities and should thus be considered in the interpretation of test scores [6, 26]. In Italy, the test most commonly used to assess confrontation naming is the Boston Naming Test (BNT), but the only currently available normative data for children relate to 160 children of different ages (from 5 years to 11 years), and thus in different school years [27].

We conducted a longitudinal investigation of confrontation naming in a nonclinical sample of Italian primary school children, testing them at the end of each school year, from the first through to the fifth grade. The aims of this study were

1. to provide longitudinal BNT data collected in a single sample of children during their primary schooling;
2. to investigate whether BNT performances are determined by socioeconomic status (SES), intelligence, age, and gender;
3. to look for possible correlations between confrontation naming and reading abilities (fluency, accuracy, and comprehension).

## 2. Method

### 2.1. Participants

As a part of a programme of early identification and treatment of learning disabilities (which had local ethics committee approval), we met the parents and teachers of 171 native Italian children attending first-grade school, from four primary schools in the northern Italian cities of Varese and Malnate. To obtain the parents informed consent to their children's participation, the aims of the study were first clearly explained to them. Children with mental retardation or other known neurological or psychiatric disorders (*n* = 3), bilingualism (*n* = 6), or whose parents withheld their consent (*n* = 3) were excluded from the study. Participant characteristics are summarized in Table 1.

### 2.2. Procedures

First of all, we collected from parents, by means of a semistructured interview, information relating to each family's socioeconomic status (SES). To assess SES, the Hollingshead Four Factor Index of Social Status was computed [28]: this measure uses education and occupation to determine a family's composite social status. The higher the index, the higher the SES. In families with both parents in employment, the scores were averaged to obtain a single score per family. On the basis of the scores obtained, the children were grouped into three categories: low (8–22), medium (23–50), and high (51–66) SES. The parents of the low SES children were mainly manual workers or in unskilled occupations or precarious employment and had a low level of education (elementary or middle school); the parents of the high SES children mainly had a high level of education (university degree) and occupied managerial positions or were in intellectual, scientific, or highly specialised professions. The parents of the children in the medium SES group mainly had an intermediate level of education (high school) and did office work or had skilled jobs in the business and services sector or technical professions.

Each child was tested individually in one 60-minute session conducted during school hours in a room set apart from the rest of the class. At the end of the first grade (May 2005), a child neuropsychiatrist and/or a psychologist individually administered a battery of standardized neuropsychological tests to assess nonverbal intelligence, confrontation naming, and reading abilities, in this order. Confrontation naming and reading abilities were assessed again at the end of the second (May 2006), third (May 2007), fourth (May 2008), and fifth (May 2009) school years.

Nonverbal intelligence was assessed using the Raven Coloured Progressive Matrices test (CPM) [29]. Each of the 36 test items consists of an incomplete abstract pattern. Participants are required to select, from a set of six, the figure needed to complete the pattern correctly. The raw scores were converted into* z*-points with reference to Italian normative data; thereafter, the* z*-points were converted into IQ scores. The reliability of the test is about 0.90.

Confrontation naming was evaluated using the 60-item revised version of the BNT and without giving any phonemic or semantic cues. The child was required to name figures shown in a book and one point was assigned for every first correct answer given within 20 seconds. There was 1 trial/stimulus for each time; no point was assigned for self-correction. Testing was discontinued after six consecutive errors [30].

Reading abilities were evaluated by means of word, pseudoword [31], and short story reading tests [32]; these tests allowed us to establish, with reference to Italian normative data for every age group, each child's reading fluency (number of syllables read per second, syll.s/sec) and reading accuracy (number of mistakes made) for each of these tasks (reading aloud), giving an overall total of six parameters. These are key parameters in transparent orthographies, like Italian. The reliability of the tests ranges from 0.752 to 0.869 for accuracy and from 0.943 to 0.967 for fluency. The results were considered poor if the parameter values were <1.5 SD (fluency) or <5th percentile (accuracy).

Reading comprehension was evaluated using Italian texts appropriate for the child's age and school year and the evaluation consisted of silent reading followed by ten multiple-choice questions. One point was given for each correct answer [32]. The reliability of the tests ranges from 0.573 to 0.700. A total score below the 25th percentile, according to Italian normative data, indicated the presence of a reading comprehension problem.

On the basis of the scores recorded in the reading tests administered at the end of the fifth school year, the children were divided into three “reading groups”: normal readers (appropriate reading fluency, accuracy, and comprehension), poor readers (at least three reading fluency and/or accuracy scores, regardless of the adequacy of their comprehension), and poor comprehenders (adequate reading fluency and accuracy, but difficulty with reading comprehension).

### 2.3. Statistical Analysis

The statistical analysis of the data was performed using the SPSS Statistics 19 package for Macintosh (IBM SPSS Statistics, Chicago, IL, USA). *p* values <0.05 were considered statistically significant.

Prior to beginning the statistical analysis, we used the Kolmogorov-Smirnov test in order to verify the normal distribution of the variables: all variables showed normal distribution except reading accuracy, reading comprehension, and SES score. Prior to multivariate analysis, SES score was converted to a dichotomous variable, low SES (8–22), and medium-high SES (23–66).

For descriptive statistics, the measures used were percentage distributions for categorical variables, and means (medians) with standard deviations (ranges) for continuous variables. Frequency distributions were compared by chi-square test and means by independent samples *t*-test, paired samples *t*-test, and one-way analysis of variance (ANOVA; Bonferroni post hoc test) for normal variables, Mann-Whitney *U* test, and Friedman test for nonnormal variables. Correlations were assessed by the Pearson's *r* (normal variables) and Spearman's rho (nonnormal variables).

General linear model with repeated measures (multivariate analysis) was used in order to verify BNT score increases from year to year and to asses which variables had a significant effect; gender, SES and nonverbal IQ were included as independent variables.

The two scores (fluency and accuracy) obtained in each reading test were reduced to a single score by means of principal component factor analysis (oblimin rotated solution); the percentage of variance explained by each analysis ranged from 68 to 75%. In addition, a further factor analysis was conducted to obtain, for each year, a single factor incorporating the parameters reading fluency, reading accuracy, and reading comprehension (the percentage of variance explained ranged from 54 to 65%).

Once their polarity had been checked, the factors thus obtained were used as dependent variables within a general linear model with repeated measures (multivariate analysis), in order to verify increases in reading abilities in each test from grade II through to grade V and also to assess which variables had a significant effect. Gender, SES, nonverbal IQ, BNT score at grade I, and reading ability at grade I were included as independent variables.

## 3. Results

Of 168 participants meeting eligibility criteria, 165 (98.2%) consented to be enrolled in the study. Among participants, 3 (1.9%) were absent at the time of the assessment, 13 (8.2%) withdrew their consent, and 17 (10.8%) changed schools. A total of 126 participants (79.2%), 68 males and 58 females, completed follow-up and were available for the analysis. The baseline characteristics of the 33 nonevaluable participants did not differ from those of other study participants (Table 1).

Demographic characteristics and assessment scores are shown in Table 2. All the children included in this study were found to have a normal nonverbal IQ (109.1 ± 9.4; range: 81–135); socioeconomic status was high in 18.3% (*n* = 23), medium in 63.5% (*n* = 80), and low in 18.3% (*n* = 23). Assessment scores obtained by the participants increased significantly with each year of education (*p* < 0.001) (Table 2).

### 3.1. Confrontation Naming Development

Significant correlations emerged between nonverbal IQ and BNT score at grades I (*r* = 0.338, *p* < 0.001), II (*r* = 0.248, *p* = 0.004), III (*r* = 0.423, *p* < 0.001), IV (*r* = 0.369, *p* < 0.001), and V (*r* = 0.402, *p* < 0.001) (Table 3).

One-way ANOVA (SES group) calculated at the end of each school year showed a highly significant SES effect from grade I to grade III (*p* < 0.001), a moderate significant effect (*p* = 0.016) at grade IV, and no effect at grade V (*p* = 0.245). Post hoc tests carried out using Bonferroni's test revealed a significantly lower number of correct answers in low SES children, while no differences were found between the medium and high SES groups (Table 4).

Longitudinal multivariate analysis confirmed that IQ (*F* = 22.560, *p* < 0.001) and SES (*F* = 4.281, *p* = 0.041) had a significant effect on the increase in correct answers from year to year (Table 5). Conversely, there emerged no gender effect (*F* = 0.183, *p* = 0.670).

### 3.2. Confrontation Naming and Reading Abilities

Correlations were found, from year to year, between confrontation naming and reading abilities, in particular comprehension (number of correct answers); BNT scores correlated better with reading fluency (syllables/second), than with reading accuracy (number of errors) (Table 3).

On the basis of the scores recorded in the reading tests administered at the end of the fifth school year, the children were divided into three “reading groups” (see Section 2.2). The BNT scores recorded by the normal readers (*n* = 112, 89.6%) were significantly higher than those of the poor readers (*n* = 4, 3.2%) and the poor comprehenders (*n* = 9, 7.2%) throughout the primary school years; instead, no significant differences were found between the poor readers and poor comprehenders (Table 6).

The multivariate longitudinal analysis (Table 7) showed that the increase in reading ability in terms of fluency and accuracy recorded from grade II to grade V, measured through the short story passage reading, was significantly influenced by the level of reading skills (fluency and accuracy) attained at the end of grade I (*F* = 60.08, *p* < 0.001) and by SES (*F* = 9.58, *p* = 0.002). When reading comprehension was also taken into consideration, the increase in passage reading skills (fluency, accuracy, and comprehension) was found to be influenced not only by the level of reading skills (fluency, accuracy, and comprehension) attained at the end of grade I (*F* = 63.27, *p* < 0.001), but also by the BNT score recorded at grade I (*F* = 8.95, *p* = 0.003), whereas there emerged no significant effect of IQ, SES, or gender.

When increase in word list reading skills (fluency and accuracy) was included as the dependent variable, the multivariate analysis showed no significant influence of the independent variables considered (IQ, BNT score, gender, and SES; data not shown). Meanwhile, improvements in pseudoword reading (fluency and accuracy) were found to be significantly influenced by reading abilities (fluency and accuracy) achieved at grade I (*F* = 72.34, *p* < 0.001).

## 4. Discussion

The BNT is a measure of word knowledge (confrontation naming), verbal learning, word retrieval, and semantic language abilities [33].

The main aim of this study was to provide normative data: our sample, particularly at grade I and grade III, recorded scores slightly higher than those recorded in another pediatrics sample [27], perhaps because we administered the BNT at the end of each school year. Also, in our sample there emerged a clear age-related improvement in BNT scores, without differences between males and females.

In our study, confrontation naming differed as a function of IQ and SES; moreover, we found a significant correlation between IQ and SES (rho = 0.355, *p* < 0.001). These results are in line with those published by other authors [34–36]. Noble et al. [35] found that SES accounted for over 30% of the variance in performance on language tasks. SES disparities between children could be mediated by aspects of their home literacy environment, degree of early print exposure, quality of early school, cognitive stimulation, nutrition, and parenting styles; moreover, a lower SES is associated with higher levels of stress as well as with changes in the function of physiological stress response systems in children and adults [37–39]. Childhood environments and experiences in different socioeconomic strata seem to be, at least in part, responsible for children's different neurocognitive outcomes, including their language ability.

What remains to be understood is the precise nature of the relationship between reading and naming. Some clues are emerging from neuroimaging studies which are beginning to uncover both commonalities and specificities in the patterns of cerebral activation recorded when participants perform the two activities [40]. Another line of research is the one followed by Nation et al. [41], who compared naming performance in two different groups of poor readers: poor decoders and poor comprehenders, with the aim of clarifying the possible relationship between naming ability and the different components of reading ability. As hypothesized by these authors, while the performance of the poor decoders was affected by word length, which is an index of phonological processing skills, that of the poor comprehenders was affected mostly by word frequency, which is an index of semantic processing ability and known to be weak in poor comprehenders [42].

In our study we found significant correlations between confrontation naming and reading abilities, in particular comprehension. Moreover, confrontation naming correlated better with reading fluency than with reading accuracy, a result that has been replicated in many studies involving RN, particularly in ones conducted in regular orthographies, in which the contribution of phonological awareness skills appears to be less relevant [18, 43, 44], and RN seems to be better able to capture the automatic aspects of reading. Correlations between reading fluency (story and word tests) showed an age-related improvement. Support for a role of confrontation naming in decoding comes from developmental studies that repeatedly report moderate correlations [45–47]: it might be assumed that the number of words added to the lexicon improves the efficiency of the direct pathway in the dual-route model of word reading. Indeed, better naming skills might be due to improved decoding skills and greater print exposure. In order to disambiguate the issue of the direction of the relationship of confrontation naming and reading improvement with age, we performed longitudinal analyses (general linear model with repeated measures), controlling for reading level achieved at the end of grade I and nonverbal IQ. We found a longitudinal relationship between confrontation naming ability and passage reading abilities (fluency, accuracy, and comprehension); this relationship did not emerge for the lists of words and pseudowords, or for the passage reading when the parameter comprehension was excluded. Furthermore, the level of reading abilities at the end of grade I was found to be a strong predictor of reading abilities, in all the tests, in the subsequent four years. The relationship between reading abilities and confrontation naming thus appears to be complex and bidirectional; furthermore, confrontation naming also appears to play a significant role in the development of text comprehension, rather than in reading fluency and accuracy. This finding is in line with the reports of several authors [48–51]. Ouellette [52] found vocabulary “depth” (semantic representations) to be a critical factor in reading comprehension performance, showing a stronger association with reading comprehension than vocabulary “breadth” (i.e., receptive vocabulary). On the basis of these findings, he suggested that vocabulary breadth is related to phonological factors, which are less relevant to reading comprehension than vocabulary depth, which taps semantic knowledge and organization. Bishop and Snowling [53] suggested that phonological impairments will place children at risk of reading difficulties early in development, when individual differences in reading are driven primarily by word recognition, whereas more general language impairments will compromise reading later on, when fluency and reading comprehension are more important. In their review, which dealt with the relationship between developmental dyslexia and specific language impairment, Bishop and Snowling [53] concluded that a child's reading profile seems to be determined by strengths and weaknesses across phonological and nonphonological (e.g. semantics and grammar) language domains, with phonological impairments impeding word recognition and nonphonological impairments limiting comprehension. Thus, “picture naming” is a multicomponential ability in which phonology, semantics, and possibly visual perception are all involved [54] playing specific roles which may be inferred from the characteristic patterns of impairment observable in the different disorders.

## Figures and Tables

**Table 1:** Demographic characteristics of participants included in the analysis and those excluded because they did not complete the follow-up. Comparisons were made by chi-square (∗), independent samples *t*-test (°), and Mann-Whitney *U* test (§). Reported data are number of patients with percentages shown in brackets, unless specified otherwise.

Participants	Included	Excluded	*p*
*N* (%)	126 (79.2)	33 (20.8)	
Gender, *N* (%)			
Male	68 (54.0)	21 (63.6)	0.319^∗^
Female	58 (46.0)	12 (36.4)	
Socioeconomic status, *N* (%)			
Low SES	23 (18.3)	6 (22.2)	0.633^∗^
Medium-high SES	103 (81.7)	21 (77.8)	
Nonverbal IQ, mean (SD)	109.1 (9.4)	107.5 (10.4)	0.375°
Confrontation naming score (correct answers) at the end of the first grade, mean (SD)	32.0 (6.6)	30.4 (8.4)	0.240°
Reading fluency (number of syllables read in one second) at the end of the first grade, mean (SD)	1.2 (0.6)	1.1 (0.8)	0.296°
Reading accuracy (number of errors) at the end of the first grade, median (range)	3.5 (0–22)	4.5 (0–18)	0.350^§^
Reading comprehension score (number of correct answers) at the end of the first grade, median (range)	8 (0–10)	7 (2–10)	**0.031** ^§^

**Table 2:** Age and assessment scores at the end of each grade of the 126 participants included in the analysis. NA = not administered because word and pseudoword reading tests are available only from the second year onwards. Paired samples *t*-tests (∗) and Friedman test (°) were used in order to verify test scores improvement from year to year.

Assessment scores	Grade	Mean	St. dev.	Median	Range	*p*
Age, years	I	6.7	0.3	6.7	6.3–7.3	—
	II	7.7	0.3	7.8	7.3–8.3	
	III	8.7	0.3	8.8	8.3–9.3	
	IV	9.8	0.3	9.8	9.3–10.3	
	V	10.7	0.3	10.7	9.3–11.3	

Nonverbal IQ	I	109.1	9.4	108	81–135	—

Confrontation naming (BNT) score (number of correct answers; total 60)	I	32.0	6.6	33	16–45	<0.001^*∗*^
	II	34.3	8.1	36	17–50	
	III	39.4	6.7	39	22–54	
	IV	43.8	6.1	45	26–56	
	V	47.9	6.0	49	29–59	

Reading fluency (number of syllables read in one second)						
Story	I	1.2	0.6	1.2	0.1–3.7	<0.001^*∗*^
	II	2.5	0.8	2.5	0.8–4.8	
	III	3.4	1.0	3.2	0.9–6.8	
	IV	4.2	1.1	4.2	1.5–7.5	
	V	3.9	1.0	3.8	1.9–7.9	
Words	I	NA	NA	NA	NA	<0.001^*∗*^
	II	2.0	0.8	1.9	0.6–3.9	
	III	2.8	0.9	2.6	1.1–5.5	
	IV	3.7	0.9	3.6	1.1–5.9	
	V	3.9	1.0	3.9	1.5–7.2	
Pseudowords	I	NA	NA	NA	NA	<0.001^*∗*^
	II	1.3	0.4	1.3	0.4–2.4	
	III	1.7	0.5	1.6	0.6–3.1	
	IV	2.1	0.6	2.0	0.9–3.6	
	V	2.3	0.7	2.2	0.8–4.5	

Reading accuracy (number of errors)						
Story	I	5.0	4.5	3.5	0–21.5	**<0.001**°
	II	5	4.4	4	0–25.5	
	III	4	3.1	3	0–15	
	IV	3.1	2.6	3	0–18	
	V	3.6	2.7	3	0–16	
Words	I	NA	NA	NA	NA	**<0.001**°
	II	6.4	5.2	5	0–27	
	III	4.6	3.8	4	0–20	
	IV	2.5	3.1	2	0–18	
	V	1.8	2.2	1	0–11	
Pseudowords	I	NA	NA	NA	NA	**<0.001**°
	II	8.4	5.7	7	0–28	
	III	6.4	4.6	6	0–23	
	IV	4.4	3.3	4	0–15	
	V	3.2	3.0	3	0–14	

Reading comprehension (number of correct answers; total 10)	I	7.6	2.1	8	0–10	**<0.001**°
	II	7.5	1.5	8	3–10	
	III	8.3	1.6	9	4–10	
	IV	8.9	1.5	10	2–10	
	V	6.9	1.9	7	0–10	

**Table 3:** Bivariate correlations between BNT, IQ, and reading abilities at each school year.

	Year	Nonverbal IQ	Story Syll.s/sec	Words Syll.s/sec	Pseudowords Syll.s/sec	Story errors	Words errors	Pseudowords errors	Comprehension
			Pearson's *r*			Spearman's rho			
Boston Naming Test scores	I	0.338^∗^	0.321^∗^	NA	NA	−0.241°	NA	NA	0.475^∗^
	II	0.248^∗^	0.309^∗^	0.155	0.020	−0.237°	−0.294°	−0.104	0.425^∗^
	III	0.423^∗^	0.457^∗^	0.282°	0.215°	−0.240°	−0.319^∗^	−0.211°	0.469^∗^
	IV	0.369^∗^	0.404^∗^	0.305^∗^	0.259°	−0.157	−0.284°	−0.207°	0.508^∗^
	V	0.402^∗^	0.419^∗^	0.386^∗^	0.235°	−0.173	−0.125	−0.219°	0.426^∗^

NA = not administered because word and pseudoword reading tests are available only from the second year onwards.

^∗^ *p* < 0.001.

°0.5 < *p* ≤ 0.001.

**Table 4:** Confrontation naming (BNT) scores obtained at the end of each grade by SES group. Comparisons were made by one-way ANOVA test (Bonferroni post hoc test).

Participants	High SES	Medium SES	Low SES	*p*
*N* (%)	23 (18.3)	80 (63.4)	23 (18.3)	
Confrontation naming (BNT) score, mean (SD)				
1st grade	34.0 (5.6)	32.9 (6.1)	26.8 (6.6)	**<0.001**
2nd grade	35.7 (6.4)	35.5 (7.9)	28.8 (8.1)	**0.002**
3rd grade	41.2 (5.7)	40.3 (6.6)	34.0 (6.1)	**<0.001**
4th grade	45.1 (5.1)	44.3 (5.9)	40.5 (6.6)	**0.016**
5th grade	48.7 (5.1)	48.1 (5.8)	45.9 (7.6)	0.245

**Table 5:** Variables associated with BNT score improvement from year to year, in multivariate general linear model with repeated measures (longitudinal analysis).

	Type III sum of squares	Mean square	*F*	*p*
Nonverbal IQ	2660.983	2660.983	22.560	**<0.001**
Low socioeconomic status	561.902	561.902	4.281	**0.041**
Male gender	23.983	23.983	0.183	0.670

**Table 6:** Boston Naming Test scores obtained by the three groups: “normal readers,” “poor readers,” and “poor comprehenders” (one-way ANOVA test, Bonferroni post hoc test).

BNT scores	Normal readers	Poor readers	Poor comprehenders	*p* value
	112 (89.6%)	4 (3.2%)	9 (7.2%)	
Year I	32.79 (6.32)	23.01 (4.97)	26.67 (5.09)	**0.001**
Year II	35.21 (7.99)	26.37 (2.89)	28.88 (7.39)	**0.019**
Year III	40.10 (6.50)	32.23 (2.08)	34.11 (6.39)	**0.010**
Year IV	44.65 (5.45)	36.33 (4.04)	38.00 (5.68)	**<0.001**
Year V	48.53 (5.67)	42.33 (2.52)	42.11 (6.17)	**<0.001**

**Table 7:** Variables associated with reading abilities improvement from grade II to grade V, in four multivariate general linear models with repeated measures (longitudinal analysis).

	Type III sum of squares	Mean square	*F*	*p*
Dependent variable: short story fluency and accuracy from grade II to grade V				
Nonverbal IQ	0.002	0.002	0.003	0.956
Low socioeconomic status	5.339	5.339	9.577	**0.002**
Male gender	0.002	0.002	0.004	0.947
BNT score at the end of grade I	0.667	0.667	1.196	0.276
Reading abilities at grade I	33.492	33.492	60.082	**<0.001**

Dependent variable: short story fluency, accuracy, and comprehension from grade II to grade V				
Nonverbal IQ	4.847	4.847	3.423	0.067
Low socioeconomic status	5.102	5.102	3.602	0.060
Male gender	1.552	1.552	1.096	0.297
BNT score at the end at Grade I	12.669	12.669	8.946	**0.003**
Reading abilities at Grade I	89.601	89.601	63.267	**<0.001**

Dependent variable: word list fluency and accuracy from grade II to grade V				
Nonverbal IQ	0.001	0.001	0.001	0.975
Low socioeconomic status	0.086	0.086	0.523	0.471
Male gender	0.020	0.020	0.121	0.728
BNT score at the end of grade I	0.001	0.001	0.002	0.961
Reading abilities at grade I	0.471	0.471	2.878	0.093

Dependent variable: pseudoword list fluency and accuracy from grade II to grade V				
Nonverbal IQ	4.784	4.784	2.440	0.121
Low socioeconomic status	0.002	0.002	0.001	0.974
Male gender	1.596	1.596	0.814	0.369
BNT score at the end of grade I	0.503	0.503	0.257	0.613
Reading abilities at grade I	141.833	141.833	72.340	**<0.001**

## References

[1] Parrila R., Kirby J. R., McQuarrie L. (2004). Articulation rate, naming speed, verbal short-term memory, and phonological awareness: longitudinal predictors of early reading development?. *Scientific Studies of Reading*.

[2] Swanson H. L., Trainin G., Necoechea D. M., Hammill D. D. (2003). Rapid naming, phonological awareness and reading: a meta-analysis of the correlational evidence. *Review of Educational Research*.

[3] Torgesen J. K., Wagner R. K., Rashotte C. A., Burgess S., Hecht S. (1997). Contributions of phonological awareness and rapid automatic naming ability to the growth of word-reading skills in second to fifth-grade children. *Scientific Studies of Reading*.

[4] Wolf M., Bowers P. G. (1999). The double-deficit hypothesis for the developmental dyslexias. *Journal of Educational Psychology*.

[5] Troia G. A., Roth F. P., Yeni-Komshian G. H. (1996). Word frequency and age effects in normally developing children's phonological processing. *Journal of Speech, Language, and Hearing Research*.

[6] Wolf M., Goodglass H. (1986). Dyslexia, dysnomia, and lexical retrieval: a longitudinal investigation. *Brain and Language*.

[7] Berninger V. W., Colwell S. O. (1985). Relationships between neurodevelopmental and educational findings in children aged 6 to 12 years. *Pediatrics*.

[8] Scarborough H. S. (1990). Very early language deficits in dyslexic children. *Child Development*.

[9] Scarborough H. S. (1998). Predicting the future achievement of second graders with reading disabilities: contributions of phonemic awareness, verbal memory, rapid naming, and IQ. *Annals of Dyslexia*.

[10] Catts H. W. (1986). Speech production/phonological deficits in reading-disordered children. *Journal of Learning Disabilities*.

[11] Dietrich J. A., Brady S. A. (2001). Phonological representations of adult poor readers: an investigation of specificity and stability. *Applied Psycholinguistics*.

[12] Wolf M. Word-wraiths: the unique contribution of the naming system to reading prediction and intervention in developmental dyslexia.

[13] Swan D., Goswami U. (1997). Picture naming deficits in developmental dyslexia: the phonelogical representations hypothesis. *Brain and Language*.

[14] Goswami U., Schneider W., Scheurich B. (1999). Picture naming deficits in developmental dyslexia in German. *Developmental Science*.

[15] Brizzolara D., Chilosi A., Cipriani P. (2006). Do phonologic and rapid automatized naming deficits differentially affect dyslexic children with and without a history of language delay? A study of Italian dyslexic children. *Cognitive and Behavioral Neurology*.

[16] Di Filippo G., Brizzolara D., Chilosi A. (2005). Rapid naming, not cancellation speed or articulation rate, predicts reading in an orthographically regular language (Italian). *Child Neuropsychology*.

[17] Di Filippo G., Brizzolara D., Chilosi A. (2006). Naming speed and visual search deficits in readers with disabilities: evidence from an orthographically regular language (Italian). *Developmental Neuropsychology*.

[18] Wimmer H., Mayringer H., Landerl K. (2000). The double-deficit hypothesis and difficulties in learning to read a regular orthography. *Journal of Educational Psychology*.

[19] Denckla M. B., Cutting L. E. (1999). History and significance of rapid automatized naming. *Annals of Dyslexia*.

[20] Savage R., Pillay V., Melidona S. (2007). Deconstructing rapid automatized naming: component processes and the prediction of reading difficulties. *Learning and Individual Differences*.

[21] Georgiou G. K., Parrila R., Papadopoulos T. C. (2008). Predictors of word decoding and reading fluency across languages varying in orthographic consistency. *Journal of Educational Psychology*.

[22] Ziegler J. C., Bertrand D., Tóth D. (2010). Orthographic depth and its impact on universal predictors of reading: a cross-language investigation. *Psychological Science*.

[23] Nation K. (2005). Picture naming and developmental reading disorders. *Journal of Research in Reading*.

[24] Hart S. A., Petrill S. A., DeThorne L. S. (2009). Environmental influences on the longitudinal covariance of expressive vocabulary: measuring the home literacy environment in a genetically sensitive design. *Journal of Child Psychology and Psychiatry, and Allied Disciplines*.

[25] Rudel R. G., Denckla M. B., Broman M., Hirsch S. (1980). Word-finding as a function of stimulus context: children compared with aphasic adults. *Brain and Language*.

[26] Bates E., Burani C., D'Amico S., Barca L. (2001). Word reading and picture naming in Italian. *Memory and Cognition*.

[27] Riva D., Nichelli F., Devoti M. (2000). Developmental aspects of verbal fluency and confrontation naming in children. *Brain and Language*.

[28] Hollingshead A. B. (1975). *Four Factor Index of Social Status*.

[29] Raven J. C. (1996). *CPM, Coloured Progressive Matrices*.

[30] Kaplan E., Goodglass H., Weintraub S. (1983). *Boston Naming Test (Revised 60-Item Version)*.

[31] Sartori G., Job R., Tressoldi P. E. (2007). *DDE-2. Batteria per la valutazione della dislessia e della disortografia evolutiva-2*.

[32] Cornoldi C., Colpo G., Gruppo M. T. (1986). *Nuove prove di lettura MT per la scuola elementare*.

[33] Halperin J. M., Healey J. M., Zeitchik E., Ludman W. L., Weinstein L. (1989). Developmental aspects of linguistic and mnestic abilities in normal children. *Journal of Clinical and Experimental Neuropsychology*.

[34] Hackman D. A., Farah M. J. (2009). Socioeconomic status and the developing brain. *Trends in Cognitive Sciences*.

[35] Noble K. G., McCandliss B. D., Farah M. J. (2007). Socioeconomic gradients predict individual differences in nerocognitive abilities. *Developmental Science*.

[36] Turkheimer E., Haley A., Waldron M., D'Onofrio B., Gottesman I. I. (2003). Socioeconomic status modifies heritability of IQ in young children. *Psychological Science*.

[37] Evans G. W. (2004). The environment of childhood poverty. *American Psychologist*.

[38] Goodman E., McEwen B. S., Dolan L. M., Schafer-Kalkhoff T., Adler N. E. (2005). Social disadvantage and adolescent stress. *Journal of Adolescent Health*.

[39] Lantz P. M., House J. S., Mero R. P., Williams D. R. (2005). Stress, life events, and socioeconomic disparities in health: results from the Americans' changing lives study. *Journal of Health and Social Behavior*.

[40] McCrory E. J., Mechelli A., Frith U., Price C. J. (2005). More than words: a common neural basis for reading and naming deficits in developmental dyslexia?. *Brain*.

[41] Nation K., Marshall C. M., Snowling M. J. (2001). Phonological and semantic contributions to children's picture naming skill: evidence from children with developmental reading disorders. *Language and Cognitive Processes*.

[42] Stothard S. E., Hulme C. (1995). A comparison of phonological skills in children with reading comprehension difficulties and children with decoding difficulties. *Journal of Child Psychology and Psychiatry and Allied Disciplines*.

[43] de Jong P. F., van der Leij A. (1999). Specific contributions of phonological abilities to early reading acquisition: results from a Dutch latent variable longitudinal study. *Journal of Educational Psychology*.

[44] Wimmer H., Mayringer H. (2002). Dysfluent reading in the absence of spelling difficulties: a specific disability in regular orthographies. *Journal of Educational Psychology*.

[45] Goswami U., Neuman S. B., Dickinson D. K. (2001). Early phonological development and the acquisition of literacy. *Handbook of Early Literacy Research*.

[46] Lee J. (2011). Size matters: early vocabulary as a predictor of language and literacy competence. *Applied Psycholinguistics*.

[47] Walley A. C., Metsala J. L., Garlock V. M. (2003). Spoken vocabulary growth: its role in the development of phoneme awareness and early reading ability. *Reading and Writing*.

[48] Ricketts J. (2011). Research review: reading comprehension in developmental disorders of language and communication. *Journal of Child Psychology and Psychiatry*.

[49] Catts H. W., Fey M. E., Tomblin J. B., Zhang X. (2002). A longitudinal investigation of reading outcomes in children with language impairments. *Journal of Speech, Language, and Hearing Research*.

[50] Nation K., Norbury C. F. (2005). Why reading comprehension fails: insights from developmental disorders. *Topics in Language Disorders*.

[51] Nation K., Snowling M. J. (2004). Beyond phonological skills: broader language skills contribute to the development of reading. *Journal of Research in Reading*.

[52] Ouellette G. P. (2006). What's meaning got to do with it: the role of vocabulary in word reading and reading comprehension. *Journal of Educational Psychology*.

[53] Bishop D. V. M., Snowling M. J. (2004). Developmental dyslexia and specific language impairment: same or different?. *Psychological Bulletin*.

[54] Johnson C. J., Paivio A., Clark J. M. (1996). Cognitive components of picture naming. *Psychological Bulletin*.

